# Exhausted signature and regulatory network of NK cells in myasthenia gravis

**DOI:** 10.3389/fimmu.2024.1397916

**Published:** 2024-09-13

**Authors:** Qing Zhang, Xingyu Han, Zhuajin Bi, Mengge Yang, Jing Lin, Zhijun Li, Min Zhang, Bitao Bu

**Affiliations:** ^1^ Department of Neurology, Tongji Hospital, Tongji Medical College, Huazhong University of Science and Technology, Wuhan, China; ^2^ Hubei Key Laboratory of Neural Injury and Functional Reconstruction, Huazhong University of Science and Technology, Wuhan, China

**Keywords:** myasthenia gravis, NK cells, IL-6, IL-21, SOCS2

## Abstract

**Introduction:**

NK cells are dysfunctional in myasthenia gravis (MG), but the mechanism is unclear. This study aims to measure associations and underlying mechanisms between the NK cells and the development of MG.

**Methods:**

Twenty healthy controls (HCs) and 53 MG patients who did not receive glucocorticoids and immunosuppressants were collected. According to the Myasthenia Gravis Foundation of America (MGFA) classification, MG patients were categorized into MGFA I group (n = 18) and MGFA II-IV group (n = 35). Flow cytometry, cell sorting, ELISA, mRNA-sequencing, RT-qPCR, western blot, and cell culture experiments were performed to evaluate the regulatory mechanism of exhausted NK cells.

**Results:**

Peripheral NK cells in MGFA II-IV patients exhibit exhausted phenotypes than HCs, marked by the dramatic loss of total NK cells, CD56^dim^CD16− NK cells, elevated PD1 expression, reduced NKG2D expression, impaired cytotoxic activity (perforin, granzyme B, CD107a) and cytokine secretion (IFN-γ). Plasma IL-6 and IL-21 are elevated in MG patients and mainly derived from the aberrant expansion of monocytes and Tfh cells, respectively. IL-6/IL-21 cooperatively induced NK-cell exhausted signature via upregulating SOCS2 and inhibiting the phosphorylation of STAT5. SOCS2 siRNA and IL-2 supplement attenuated the IL-6/IL-21-mediated alteration of NK-cell phenotypes and function.

**Discussion:**

Inhibition of IL-6/IL-21/SOCS2/STAT5 pathway and recovery of NK-cell ability to inhibit autoimmunity may be a new direction in the treatment of MG.

## 1 Introduction

Myasthenia gravis (MG) is an antibody-mediated autoimmune disease affecting neuromuscular transmission, characterized clinically by recurrent episodes of muscle weakness (1). Natural killer (NK) cells are an important element of innate immunity, however, their role in the development of MG remains unclear (2, 3). Our previous study indicated patients with generalized MG exhibited NK cell depletion, characterized by a lower absolute count and percentage of NK cells (4). Restoring NK cell-mediated inhibition of autoimmune processes is a valuable direction related to the treatment of MG (5).

NK cells predominantly exhibit cytotoxic activity and immunoregulation effects and act as a double-edged sword in autoimmune diseases (6). NK cells may help control type 1 diabetes (TID) and multiple sclerosis by killing autoreactive immune cells (7, 8). However, NK cells contribute to the development of idiopathic inflammatory myopathy and systemic lupus erythema1tosus (SLE) under pathological conditions by producing IFN-γ and activating B cells (9, 10).

Similar to other autoimmune conditions (11), MG patients suffer a significant decline in the number, cytotoxicity, and cytokine production of NK cells, but the regulatory mechanism of NK cells in MG remains poorly described (2). Yang et al. found that the adoptive transfer of NK cells can effectively relieve the symptoms of experimental autoimmune myasthenia gravis (EAMG) by inhibiting follicular helper (Tfh) cells and germinal center B cells (12). NK subpopulations are closely associated with both clinical progression and therapeutic efficacy in patients with MG (3, 13). Although several studies have verified that inflammation and the cytokine storm are the main causes of NK-cell dysfunction in diverse pathological conditions (14, 15), the mechanism of regulating NK cell activity during MG development remains largely unknown.

In this study, MG patients who did not use glucocorticoids and immunosuppressants were enrolled to characterize the NK-cell dysfunction with different disease states. *In vitro* experiments and transcriptomic sequencing were conducted to explore molecular regulatory networks of NK cells, which may provide novel ideas for further application of NK-cell therapy in MG treatment.

## 2 Materials and methods

### 2.1 Participants and sample collection

This study included 53 MG patients who were admitted to the Department of Neurology, Tongji Hospital, Tongji Medical College, Huazhong University of Science and Technology from May 2020 to December 2023. Samples from 20 age- and gender-matched healthy volunteers were included as controls. Inclusion criteria are as follows: (1) confirmed diagnosis of MG based on characteristic fatigable weakness with the presence of acetylcholine receptor antibody (AchR-Ab) (16); (2) immunotherapy-naïve or discontinued glucocorticoids and immunosuppressants at least 6 months (17); (3) aged 18 to 80 years old. Exclusion criteria were other autoimmune diseases, malignancies (except thymoma), or severe infections at admission. According to the Myasthenia Gravis Foundation of America (MGFA) classification (18), MG patients were categorized into MGFA I group and MGFA II-IV group. MGFA classification I is defined as weakness restricted to ocular muscles and MGFA classification II-IV were categorized as mild, moderate and heavy bulbar, respiratory and/or generalized weakness, respectively.

Heparinized blood was drawn in the morning the day after admission. Plasma was extracted by centrifugation of whole blood and stored at −80°C until analysis. Peripheral blood mononuclear cells (PBMCs) were isolated by Lymphoprep gradient centrifugation (Catalog No. 07801, STEMCELL Technologies, Canada) and used directly or cryopreserved as previously described (19). Cryopreserved PBMCs were thawed as outlined elsewhere (20) and initially stained using Fixable Viability Stain 700 (Catalog No. 564997, BD Pharmingen, San Diego, USA) for the exclusion of dead cells.

To detect major immune cell subsets, surface proteins were stained with the anti-human antibodies: CD3 APC-H7 (Clone SK7, Catalog No. 560176)/CD4 FITC (Clone L200, Catalog No.550628)/CD14 Horizon V450 (Clone M5E2, Catalog No. 561390)/CD56 BV510 (Clone NCAM16.2, Catalog No. 563041) (BD Pharmingen, San Diego, USA) and CD8 PerCP (Clone SK1, Catalog No. 344707)/CD19 BV650 (Clone HIB19, Catalog No. 302237)/CXCR5 APC (Clone J252D4, Catalog No. 356907)/PD-1 BV605 (Clone NAT105, Catalog No. 367425)(BioLengend, San Diego, USA). For intracellular staining, PBMCs were incubated with Leuko Act Cktl with GolgiPlug (Catalog No. 550583, BD Pharmingen, San Diego, USA) for 4 h at 37°C followed by surface marker staining. The representative gating strategy of major immune cell subsets is provided in **Supplementary Figure S1**. After fixation and permeabilization using the Cytofix/Cytoperm Soln kit (Catalog No. 554715, BD Pharmingen, San Diego, USA), intercellular IL-6 PE Dazzle™ 594 (Clone MQ2-13A5, Catalog No. 501121)/IL-21 PE (Clone 3A3-N2, Catalog No. 513003)(BioLengend, San Diego, USA) were stained.

NK surface protein staining was performed using the multiple conjugated anti-human antibodies: CD3 APC-H7 (Clone SK7, Catalog No. 560176)/CD16 FITC (Clone 3G8, Catalog No. 555406)/CD56 BV510 (Clone NCAM16.2, Catalog No. 563041)/NKp46 BV421 (Clone 9E2, Catalog No. 564065)/NKG2A BV786 (Clone 131411, Catalog No.747917)/NKG2D PE (Clone 1D11, Catalog No. 557940) (BD Pharmingen, San Diego, USA) and FASL BV650 (Clone NOK-1, Catalog No. 744100)/PD-1 BV605 (Clone NAT105, Catalog No.367425)/ICOS PE-Cy7 (Clone C398.4A, Catalog No. 313519) (BioLengend, San Diego, USA). Functional NK cell response staining: PBMCs were stimulated with Leuko Act Cktl with GolgiPlug, stained CD3 APC-H7/CD16 FITC/CD56 BV510, permeabilized, and then co-stained with the intracellular CD107a APC (Clone H4A3, Catalog No. 560664)/IFN-γ PE-CY7 (Clone B27, Catalog No. 557643) (BD Pharmingen, San Diego, USA) and perforin PE (Clone dG9, Catalog No. 308105)/granzyme B BV421(Clone QA18A28, Catalog No. 396413)/IL-10 PE-Dazzle™ 594 (Clone JES3-9D7, Catalog No. 501425) (BioLengend, San Diego, USA).

Flow cytometric data was acquired with CytoFLEX flow cytometer (Beckman Coulter, Fullerton, USA) and analyzed using Flowjo 10.0 (Tree Star, Ashland, USA). Unsupervised analyses were conducted using t-distributed stochastic neighbor embedding (t-SNE) to further screen the subpopulation changes (21). FMO (fluorescence minus one) was used as the gating control.

For cell sorting experiments, PBMCs were stained with Fixable Viability Stain 700, CD3 APC-H7, and CD56 BV510. Sorting procedures were performed on a MoFlo XDP Cell Sorter (Beckman Coulter, Fullerton, USA). Gating strategies for major NK subsets and sorting NK cells are presented in **Supplementary Figures S2A, B**.

### 2.2 NK-cell cytotoxicity assay

K562 cells (Catalog No. CX0029, Boster, Wuhan, China) were used as the target cells and were labeled with DiOC18 (Catalog No. 34215-57-1, Maokangbio, Shanghai, China). Isolated NK cells were taken as effector cells and co-cultured with labeled K562 cells at effector to target (E/T) ratio 4:1, 2:1, and 1:1 for 12 h at 37°C (5% CO2). Live/reaming live target cells were applied using Fixable Viability Stain 780 (Catalog No. 565388, BD Pharmingen, San Diego, USA) and then detected with the flow cytometer. The percentage of killing by NK cells was calculated by the equation (22):


$$Killing\%=\frac{Reaming\;live\;K562\%\left(E:T\;ratio=n:1\right)-\;Reaming\;live\;K562\%\left(E:T\;ratio=0:1\right)}{100\%-Reaming\;live\;K562\%\left(E:T\;ratio=0:1\right)}$$

### 2.3 Enzyme-linked immunosorbent assay (ELISA)

The plasma samples were melted at room temperature before the experiment. Cytokine concentrations of IL-2 (Catalog No. HM10211), IL-6 (Catalog No. HM10205), IL-12 p70 (Catalog No. HM10390), IL-15 (Catalog No. HM10938), IL-18 (Catalog No. HM10337), and IL-21 (Catalog No. HM11908) were measured by using Human Quantikine ELISA Kit (Bioswamp, Wuhan, China). The absorbance at 450 nm was measured with Microplate Reader (Thermo Fisher, Waltham, USA).

### 2.4 *In vitro* co-culture of NK cells

Previous studies have shown the dependent inhibition of NK cells using IL-6 (23) and IL-21 (24) without side effects, so we chose the same intervention condition. Sorted NK cells were treated with IL-6 (100 ng/mL, Catalog No. HY-P7044) and/or IL-21 (50 ng/mL, Catalog No. HY-P7038) (MedChemExpress, Princeton, USA) for 72 h. Apoptosis assay was performed by Annexin V-PE/7-AAD apoptosis detection kit (Catalog No. 40310ES20, Yeasen, Shanghai, China). NK-cell phenotypes and function were detected by flow cytometry as above. Sorted NK cells were expanded with an NK Cell Robust Expansion kit (Catalog No. CT-001, Stemery, Fuzhou, China) according to the manufacturer’s instructions and used for further experiments.

### 2.5 RNA-sequencing

Total mRNA from the isolated NK cells was extracted by TRIzol and sequenced using the BGISEQ platform (Huada Gene Technology Co. Ltd., Wuhan, China). RNA quality was verified with an Agilent 2100 Bioanalyzer (Agilent Technologies, Santa Clara, USA). Raw sequencing data was uploaded to the NCBI database (Submission ID: SUB13951594, BioProject ID: PRJNA1035806). The subsequent analysis and graphing were conducted in Dr. Tom analysis system (https://biosys.bgi.com). Differential transcripts with |log2FC| > 1 and adjusted P-value < 0.05 were selected as differentially expressed genes (DEGs). Next, we conducted the Gene Ontology (GO) and Kyoto Encyclopedia of Genes and Genomes (KEGG) analyses for functional annotation of DEGs. Volcano plots and enrichment plots were generated using the R package.

### 2.6 Real-time quantitative PCR analysis (RT-qPCR)

Expanded NK cells were treated with IL-6 and/or IL-21 as described above. Cells were harvested and mRNA was extracted by TRIzol (Catalog No. 15596026, Invitrogen, Carlsbad, USA). cDNA was prepared using PrimeScript^TM^ RT Reagent Kit (Catalog No. RR037B, Takara, Shiga, Japan). GAPDH was used as an internal control, RT-qPCR of S100 calcium binding protein A8/9 (S100A8/9), four-and-a-half domain protein 3 (FHL3), immunoglobulin J chain (JCHAIN), suppressor of cytokine signaling 2 (SOCS2), and Fc fragment of IgM receptor (FCMR) was performed by SYBR-Green Kit (Catalog No. 11201ES08, Yeasen, Shanghai, China). Expression of selected genes was measured using CFX Connect Real-Time System (BioRad Laboratories, Hercules, USA) and illustrated by the *2^−(ΔΔCt)^* method. The primers used in this study are shown in **Supplementary Table S1**.

### 2.7 Western blot analysis

Expanded NK cells were homogenized in RIPA lysis buffer with PMSF (100:1, Catalog No. P0013B, Catalog No. ST2573, Beyotime, Beijing, China). The proteins were transferred to PVDF membranes. Then the membranes were incubated with primary specific antibodies: rabbit anti-SOCS2 (1:1000, Catalog No. A9190, ABclonal, Wuhan, China), rabbit anti-STAT5A/B (1:2000, Catalog No.13179-1-AP, Proteintech, Chicago, USA), rabbit anti-Phospho-STAT5-Y694 (1:500, Catalog No. AP0887, ABclonal, Wuhan, China), and rabbit anti-β-actin (1:2000, Catalog No. 20536-1-AP, Proteintech, Chicago, USA). Then the membranes were incubated with HRP-conjugated goat anti-rabbit secondary antibody (1:5000, Catalog No. SA00001-2, Proteintech, Chicago, USA). Target protein bands were detected with chemiluminescence reagents and visualized using ImageJ 1.5(NIH, Maryland, USA).

### 2.8 Small interfering RNA (siRNA) transfection and drug treatments

According to the manufacturer’s instruction, the isolated NK cells were respectively transfected with siSOCS2 and negative control (NC) using Lipo2000 (Catalog No. 11668030, Invitrogen, Chicago, USA). The siRNA sequences were 5’- GAAGGAACTTTCTTGATTA-3’ (siSOCS2) and 5’-UUCUCCGAACGUGUCACGU’ (NC). 48 h after transfection, the NK cells were subjected to stimulation with IL-6/IL-21 (100 ng/mL/50 ng/mL) and/or STAT5 activator IL-2 (20 ng/mL, Catalog No. HY-P7037, MedChemExpress, Princeton, USA) for 72 h *in vitro*. Cells were harvested and collected for flow cytometry analysis.

### 2.9 Statistical analysis

Continuous variables are represented by mean ± SD or median [IQR], whereas categorical variables are presented as n (%). Differences between groups were analyzed using chi-squared, Mann-Whitney, Kruskal-Wallis tests, or one-way ANOVA, as appropriate. Spearman’s correlation analysis was performed to detect the correlation. The correlation coefficient |r| > 0.5 was considered a strong correlation. Two-sided statistical significance was defined as P values < 0.05 after Bonferroni corrections for multiple comparisons. Statistical analyses were conducted using SPSS 24.0 (IBM, Armonk, USA), Origin 2021 (OriginLab, Northampton, USA), and GraphPad Prism 8.01 (GraphPad Prism, San Diego, USA).

## 3 Results

### 3.1 Clinical characteristics

The overall subjects were divided into healthy control (HC) group (n = 20), MGFA I group (ocular MG, n = 18), and MGFA II-IV group (generalized MG, n = 35). AChR-Ab titers were significantly lower in the MGFA I group than in the MGFA II-IV group. There were no statistically significant differences in other clinical variables between the groups and the general clinical data are summarized in **Table 1**.

**Table 1:** Characteristics of subjects enrolled in the study.

	HC (n = 20)	MGFA I (n = 18)	MGFA II-IV (n = 35)	P
Age (yr), mean ± SD	47.25 ± 17.58	49.79 ± 16.62	47.56 ± 16.73	0.864
Sex, n (%)				
Female	11 (55.0%)	10 (55.6%)	19 (54.3%)	0.996
Male	9 (45.0%)	8 (44.4%)	16 (45.7%)	
Disease duration (yr), median [IQR]	–	0.33 [0.09 – 2.50]	0.50 [0.25-2.35]	0.488
Imaging diagnosis for thymus, n (%)				
Normal	–	14 (77.8%)	17 (48.6%)	0.110
Hyperplasia	–	2 (11.1%)	8 (22.9%)	
Thymoma	–	2 (11.1%)	10 (28.6%)	
MG subgroups				
EOMG	–	8 (44.4%)	20 (57.1%)	0.380
LOMG	–	10 (55.6%)	15 (42.9%)	
AChR-Ab (nmol/L), mean ± SD	–	4.58 [2.30 – 7.90]	8.63 [4.80 – 13.07]	0.012*
Repetitive nerve stimulation, n (%)				
Positive	–	6 (33.3%)	19 (54.2%)	0.102
Negative	–	6 (33.3%)	4 (11.4%)	
Treatment, n (%)				
Pyridostigmine	-	4 (22.2%)	13 (37.1%)	0.270
Drug-free	-	13 (76.5%)	22 (62.9%)	

AChR-Ab, acetylcholine receptor antibody; MGFA, Myasthenia Gravis Foundation of America; EOMG, early onset MG; LOMG, late onset MG. *, P <0.05.

### 3.2 Changes of major immune cell subsets in MG

**Figure 1:**
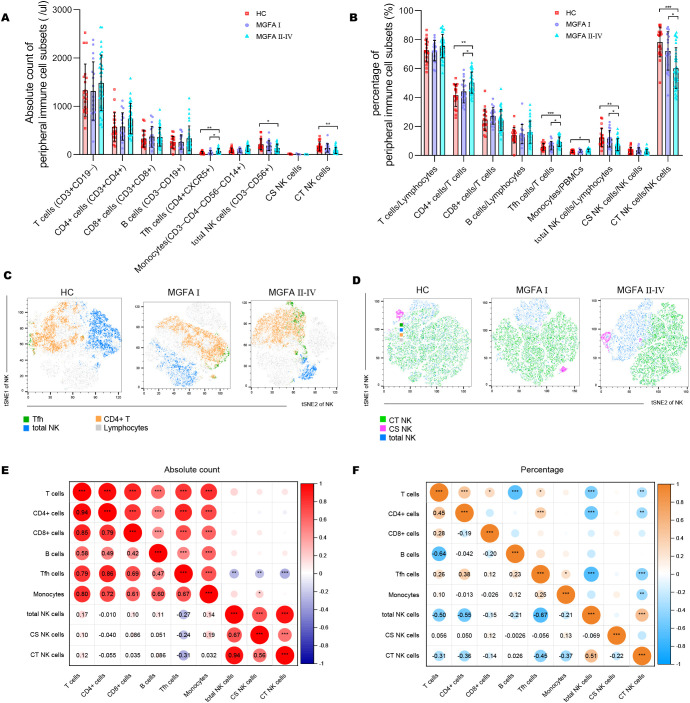
Identification of major immune cell subsets in myasthenia gravis patients. **(A, B)** Absolute counts and percentages of major immune cell subsets between HC (n = 20), MGFA I (n = 18), and MGFA II-IV (n = 35) group. **(C, D)** t-SNE analysis on Tfh cells, NK subsets performed separately by HC, MGFA, and MGFA II-IV group. **(E, F)** Correlation analysis of absolute count and the percentage between the major immune cell subsets for all subjects (n = 73). The numbers in the lower left part of the heatmap show the values of the correlation coefficient. HC, healthy control; MGFA, Myasthenia Gravis Foundation of America. Data are presented as mean ± SD. *P < 0.05, **P < 0.01, ***P < 0.001.

The NK-cell population in MG patients has been classically divided into cytotoxic CD56^dim^CD16+ NK (CT NK) cells and cytokine-secreting CD56^bright^CD16− NK (CS NK) cells (25). The absolute counts of Tfh cells were elevated in MGFA II-IV group compared to HC group, whereas total NK cells, CT NK cells were decreased (**Figure 1A**). As shown in **Figure 1B**, the percentages of CD4+ cells and Tfh cells were significantly higher in MGFA II-IV group than in MGFA I group and HC group, whereas total NK cells and CT NK cells were lower. Consistent with the results in **Figure 1B**, the t-SNE analysis revealed Tfh expansion and NK-cell depletion in MG (**Figures 1C, D**). The decrease in total NK counts was strongly correlated with the reduction of CT NK cells and CS NK cells (**Figure 1E**). The lower percentage of total NK cells was strongly correlated with the decreased CT NK cells and expanded CD4+ cells and Tfh cells (**Figure 1F**).

### 3.3 Exhausted signature of NK cells in MG

**Figure 2:**
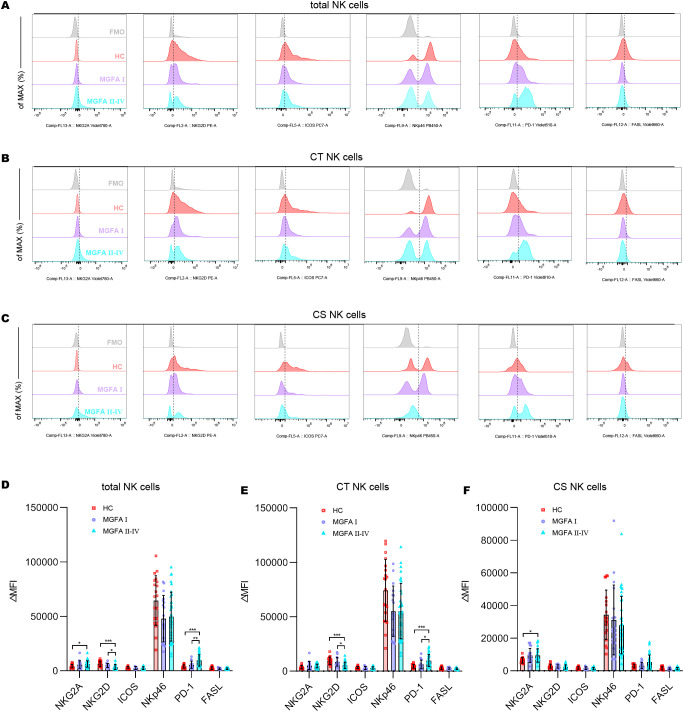
Analysis of receptor expression on NK-cell subsets in myasthenia gravis patients. **(A–C)** Representative flow histograms showing the ΔMFI of NKG2A, NKG2D, ICOS, NKp46, PD1, and FASL on total NK cells, CT NK cells, CS NK cells between HC (n = 20), MGFA I (n = 18), and MGFA II-IV (n = 35) group. **(D–F)** Summary dot plots showing the ΔMFI values of NKG2A, NKG2D, ICOS, NKp46, PD1, and FASL on NK-cell subsets between the three groups. ΔMFI value was calculated by subtracting the MFI value of the FMO control. HC, healthy control; MGFA, Myasthenia Gravis Foundation of America; CT NK, cytotoxic CD56^dim^CD16+ NK; CS NK, cytokine-secreting CD56^bright^CD16− NK; MFI, median fluorescence intensity; FMO, fluorescence minus one control. Data are presented as mean ± SD. *P < 0.05, **P < 0.01, ***P < 0.001.

Representative flow histograms showing the ΔMFI (median fluorescence intensity) of surface receptors on NK subsets are shown in **Figures 2A–C**. The ΔMFI value of NKG2A on both total NK cells and CS NK cells was elevated in MGFA II-IV group compared to HC group. PD1 on total NK cells and CT NK cells was significantly higher in MGFA II-IV group than in MGFA I group and HC group, whereas the NKG2D on total NK cells and CT NK cells were lower. No expression differences in ICOS, NKp46, and FASL were found between the three groups (**Figures 2D–F**).

**Figure 3:**
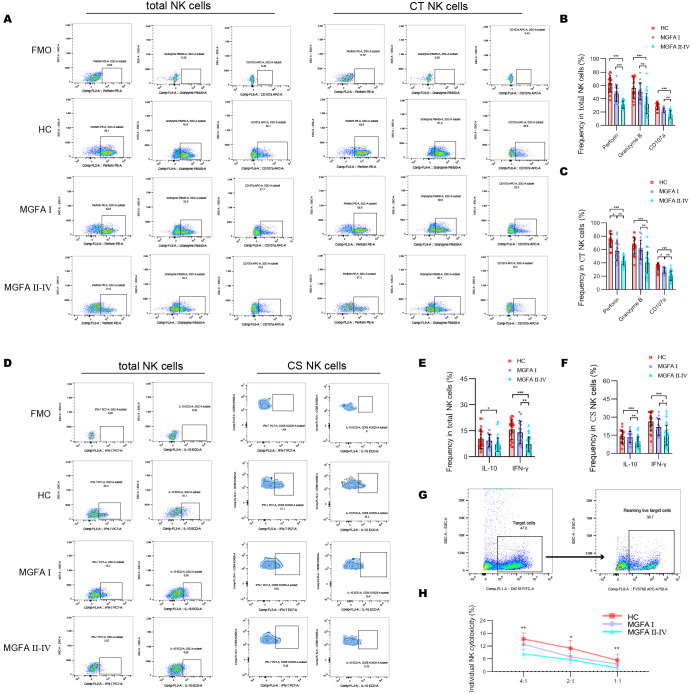
Assessment of NK-cell cytotoxicity and cytokine-secretion in myasthenia gravis patients. **(A–C)** Representative flow cytometry plots and summary dot plots showing the frequency of perforin, granzyme B, and CD107a production by total NK cells and CT NK cells between HC (n = 20), MGFA I (n = 18), and MGFA II-IV (n = 35) group. **(D–F)** Representative flow cytometry plots and summary dot plots showing the frequency of IFN-γ and IL-10 secretion by total NK cells and CS NK cells between the three groups. **(G)** Representative flow cytometry plots distinguished target cells and reaming live target cells. **(H)** Quantification of the percentage of the killing of K562 cells by NK cells between the three groups. HC, healthy control; MGFA, Myasthenia Gravis Foundation of America; CT NK, cytotoxic CD56^dim^CD16+ NK; CS NK, cytokine-secreting CD56^bright^CD16− NK. Data are presented as mean ± SD. *P < 0.05, **P < 0.01, ***P < 0.001.

The positive expressions of cytotoxic molecules, including perforin, granzyme B, and CD107a in total NK cells were significantly decreased in MGFA II-IV patients than in HCs (healthy controls) and MGFA I patients. Likewise, MGFA II-IV group revealed decreased perforin, granzyme B, and CD107a expression in CT NK cells than the other groups (**Figures 3A–C**). Cytokine secretion analysis demonstrated that the percentage of IL-10 and IFN-γ in total NK cells and CS NK cells in the MGFA II-IV group was significantly lower than in the HC group. Compared to MGFA I patients, a reduction in frequencies of IFN-γ–producing total NK cells and CS NK cells was observed in MGFA II-IV patients (**Figures 3D–F**). To further determine NK-cell cytotoxicity, we evaluate the NK cell-mediated cytotoxicity against K562 cell line. As shown in **Figures 3G, H**, NK killing ability against K562 was gradually impaired among the three groups.

### 3.4 Changes of cytokine profiles in MG

**Figure 4:**
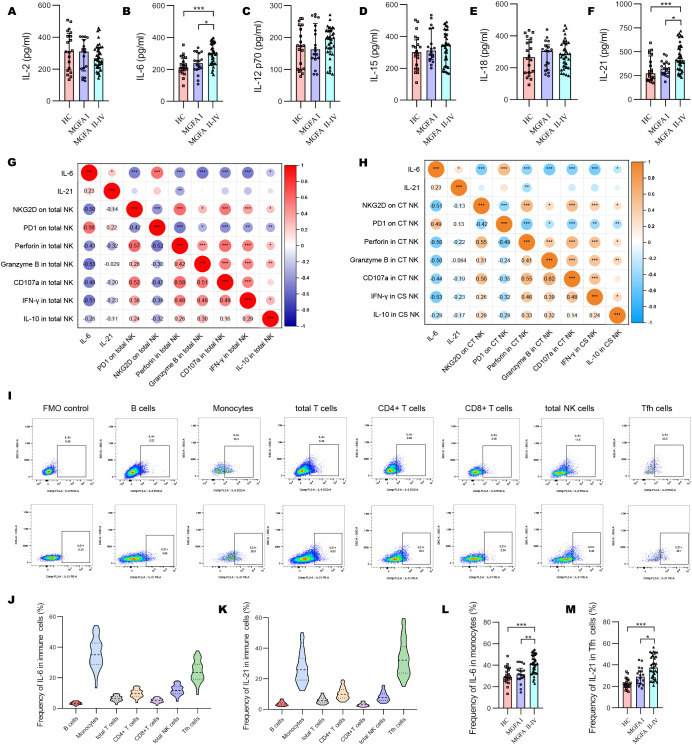
Changes of plasma cytokines in myasthenia gravis patients. **(A–F)** Levels of plasma IL-2, IL-6, IL-12 p70, IL-15, IL-18, IL-21 between HC (n = 20), MGFA I (n = 18), and MGFA II-IV (n = 35) group. **(G)** Correlation analysis between plasma IL-6, IL-21, and expression of NKG2D, PD1, perforin, granzyme B, CD107a, IFN-γ, and IL-10 in total NK cells for all subjects (n = 73). **(H)** Correlation analysis between plasma IL-6, IL-21 and expression of NKG2D, PD1, perforin, granzyme B, CD107a, IFN-γ, and IL-10 in CT NK cells and CS NK cells for all subjects. The numbers in the lower left part of the heatmap show the values of the correlation coefficient. **(I–K)** Representative flow cytometry plots and summary violin plots showing the frequency of IL-6 and IL-21 secretion by major immune cells for all subjects. **(L, M)** Summary dot plots showing the frequency of IL-6+ monocytes and IL-21+ Tfh cells between HC, MGFA I, and MGFA II-IV group. HC, healthy control; MGFA, Myasthenia Gravis Foundation of America; CT NK, cytotoxic CD56^dim^CD16+ NK; CS NK, cytokine-secreting CD56^bright^CD16− NK. Data are presented as mean ± SD. *P < 0.05, **P < 0.01, ***P < 0.001.

The concentrations of IL-6 and IL-21 increased with the progression of MG disease (**Figures 4A–F**). We also compared the plasma levels of IL-2, IL-12 p70, IL-15, and IL-18 across the groups but observed no significant difference. As shown in **Figure 4G**, IL-6 revealed a strong positive association with PD1, and negative associations with NKG2D, granzyme B and IFN-γ expression in total NK cells. In addition, IL*-*6 strongly negatively correlated with the levels of NKG2D in CT NK cells and IFN-γ in CS NK cells (**Figure 4H**). Next, we investigated the origin of IL-6 and IL-21 secretion in the major immune cell subsets. The most significant source of IL-6 and IL-21 were macrophages and Tfh cells, respectively (**Figures 4I–K**). IL-6–producing monocytes and IL-21–producing Tfh cells were significantly higher in MGFA II-IV group than in MGFA I group and HC group (**Figures 4L, M**).

### 3.5 Synergy effect of IL-6 and IL-21 on NK-cell exhaustion

**Figure 5:**
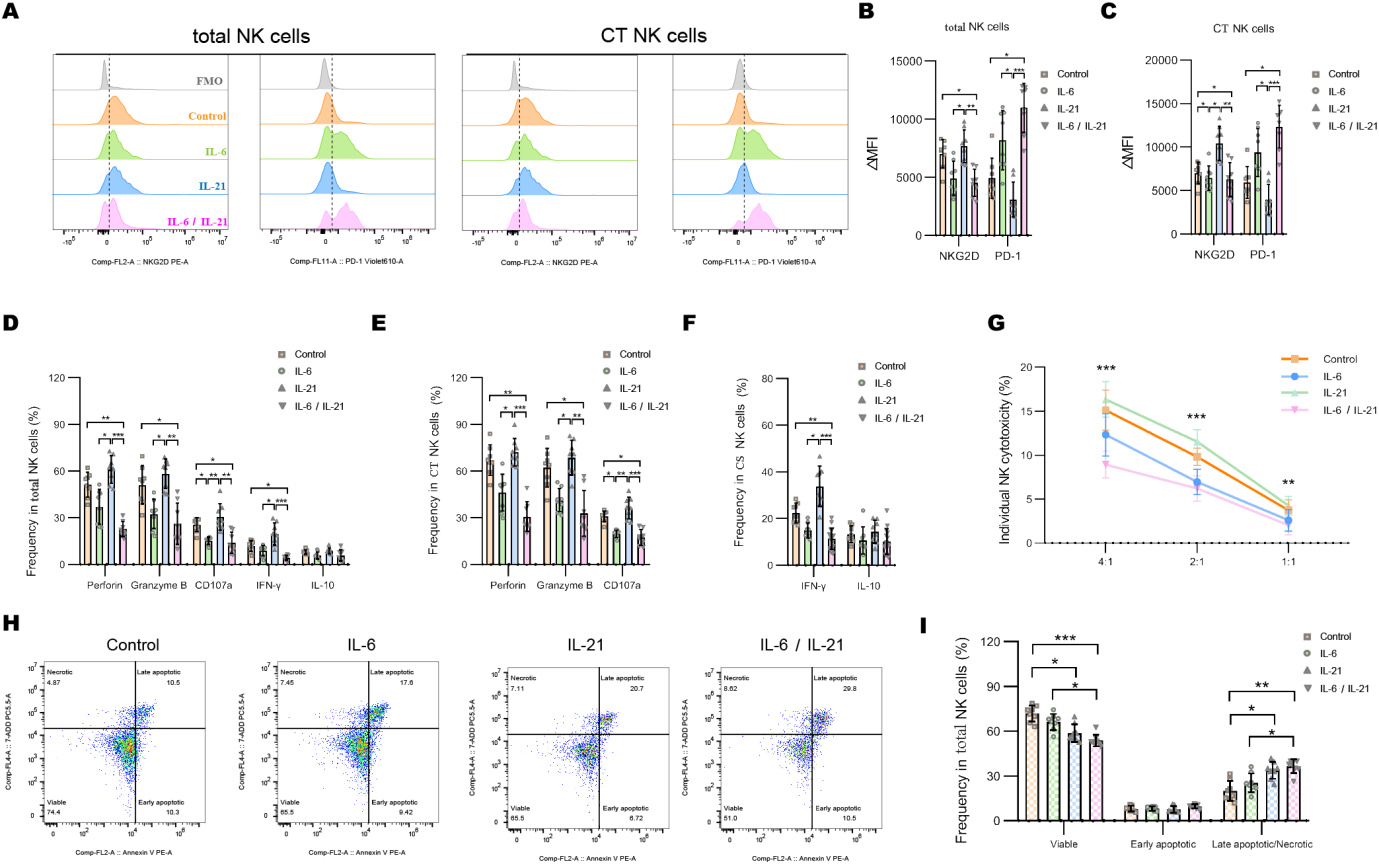
IL-6 and IL-21 induced the exhaustion of NK cells *in vitro*. **(A–C)** Representative flow histograms and summary dot plots showing the ΔMFI of NKG2D and PD1 on total NK cells, CT NK cells between control, IL-6, IL-21, and IL-6/IL-21 group. **(D–F)** Summary dot plots showing the frequency of perforin, granzyme B, CD107a, IFN-γ, and IL-10 production by NK-cell subsets between the four groups. **(G)** Quantification of the percentage of the killing of K562 cells by NK cells between the four groups. **(H, I)** Representative flow cytometry plots and summary dot plots representing the percentage of viable (Annexin V−/7-ADD−), early apoptotic (Annexin V+/7-ADD−), and late apoptotic (Annexin V+/7-ADD+)/necrotic (Annexin V-/7-ADD+) NK cells. ΔMFI value was calculated by subtracting the MFI value of the FMO control. MFI, median fluorescence intensity; CT NK, cytotoxic CD56^dim^CD16+ NK; FMO, fluorescence minus one control. Experiments were measured for eight blood NK-cell samples. Data are presented as mean ± SD. *P < 0.05, **P < 0.01, ***P < 0.001.

Representative flow dot-plots demonstrating NK-cell cytokine secretion and cytotoxic function after IL-6 and/or IL-21 treatment are shown in **Supplementary Figures S3**, **S4**. Compared to the IL-21 (50 ng/mL, 72h) stimulation, IL-6 alone (100 ng/mL, 72h) is able to decrease the ΔMFI of NKG2D and increase PD1 on total NK cells and CT NK cells. The combination of IL-6/IL-21 exhibited a stronger effect on NKG2D and PD1 expression than IL-6 alone (**Figures 5A–C**). IL-6 stimulation showed weaker inhibition of cytotoxicity (perforin, granzyme B, CD107a production) and secretion of IFN-γ than control. The combined inhibitory effect of IL-6/IL-21 was stronger (**Figures 5E, F**). Similarly, NK cell-mediated lysis of K562 cells was reduced significantly by IL-6/IL-21 stimulation (**Figure 5G**). IL-21 was found to have an activating effect on NK cells, increasing NKG2D expression, cytotoxicity, IFN-γ section, and instead decreased PD1 expression, although this difference was not statistically significant. Nevertheless, IL-21 alone could induce apoptosis and necrosis to curtail the life span of NK cells. Under synergistic stimulation of IL-6 and IL-21, more NK cells underwent apoptosis and necrosis than control (**Figures 5H, I**).

### 3.6 IL-6/IL-21 constrains NK-cell activity via SOCS2/STAT5 *in vitro*

**Figure 6:**
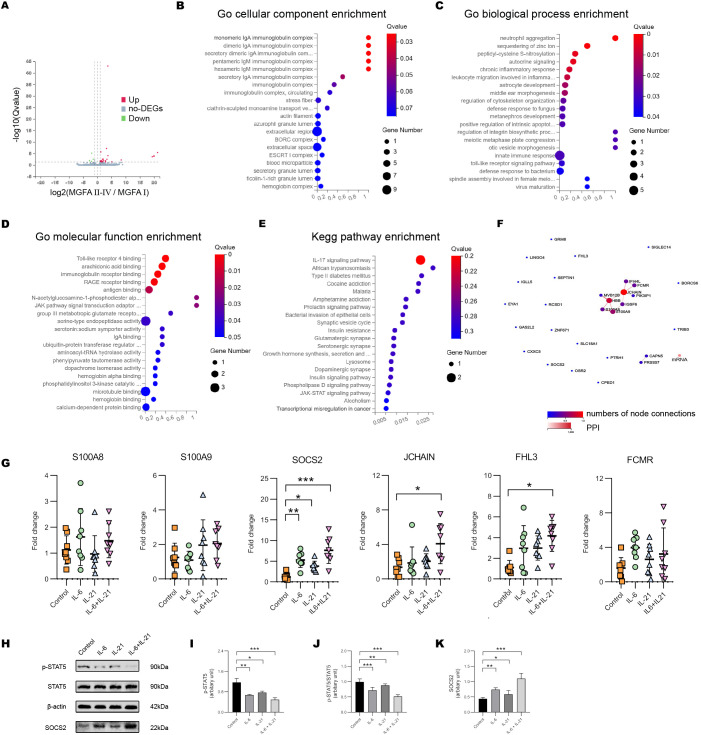
IL-6/IL-21 regulates SOCS2/STAT5 signaling in NK cells. **(A)** Volcano plot showing the DEGs between MGFA I (n = 3) and MGFA II-IV (n = 4) group. **(B–D)** GO enrichment analysis of the DEGs in cellular components, biological process, and molecular function. **(E)** KEGG enrichment pathway analysis of the DEGs. **(F)** Protein-protein interaction network for DEGs. **(G)** The mRNA expression of S100A8/9, SOCS2, JCHAIN, FHL3, and FCMR in NK cells between control, IL-6, IL-21, and IL-6/IL-21 group. **(H–K)** Protein bands and levels of p-STAT5, p-STAT5/STAT5, and SOCS2 between the four groups. MGFA, Myasthenia Gravis Foundation of America. RT-qPCR was measured for eight blood NK-cell samples and western blot was measured for five samples. Data are presented as mean ± SD. *P < 0.05, **P < 0.01, ***P < 0.001.

We further assessed the gene expression pattern of NK cells by BGISEQ sequencing platform with high throughput. Compared to the MGFA I group, 27 genes were up-regulated and 4 genes were down-regulated in MGFA II-IV group (**Figure 6A**). GO analysis identified that the most enriched terms of cellular components, biological process and, molecular function were IgA immunoglobulin complex, neutrophil aggregation, TLR4 binding, respectively (**Figures 6B–D**). For KEGG analysis, enriched pathways included IL-17 signaling pathway, African trypanosomiasis, type II diabetes, etc (**Figure 6E**). Protein−protein interaction network of hub genes is shown in **Figure 6F**. We screened the six previously documented DEGs implicated in the regulation of NK cells to further corroborate our findings.

When co-stimulated with IL-6/IL-21, the mRNA levels of SOCS2, JCHAIN, and FHL3 in NK cells were increased. The transcript level of SOCS2 was more distinct which could be elevated with IL-6 alone or IL-21 alone stimulation (**Figure 6G**). SOCS2 is driven by the cytokine-mediated activation of JAK/STAT signaling, inhibiting STAT5 phosphorylation and activation via a negative feedback loop (26). The IL-6/IL-21 synergistic group exhibited a significant upregulation of SOCS2 and downregulation of pSTAT5 and pSTAT5/STAT5 protein levels, surpassing that observed in the stimulus-alone group. (**Figures 6H–K**).

**Figure 7:**
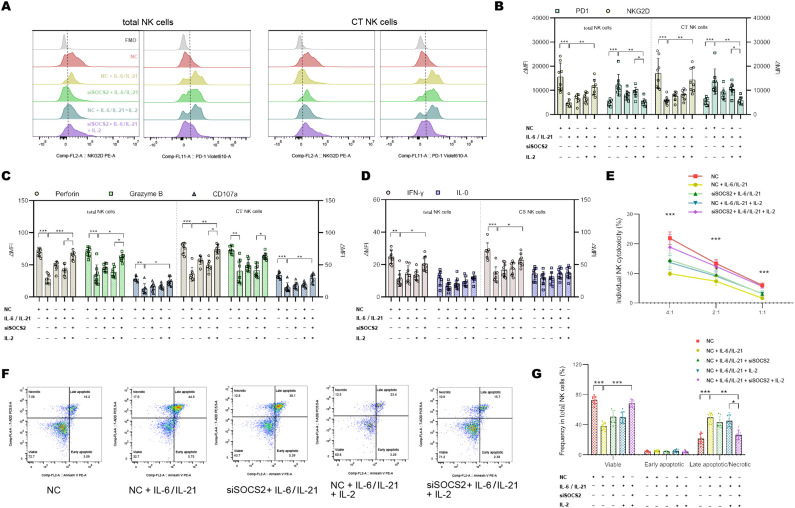
siSOCS2/IL-2 restored NK phenotypes and function *in vitro*. **(A, B)** Representative flow histograms and summary dot plots showing the ΔMFI of NKG2D and PD1 on total NK cells, CT NK cells between NC, NC + IL-6/IL-21, siSOCS2 + IL-6/IL-21, NC + IL-6/IL-21 + IL-2, siSOCS2+ IL-6/IL-21 + IL-2 group. **(C, D)** Summary dot plots showing the frequency of perforin, granzyme B, CD107a, IFN-γ, and IL-10 production by NK-cell subsets between the five groups. **(E)** Quantification of the percentage of the killing of K562 cells by NK cells between the five groups. **(F, G)** Representative flow cytometry plots and summary dot plots representing the percentage of viable (Annexin V−/7-ADD−), early apoptotic (Annexin V+/7-ADD−), and late apoptotic (Annexin V+/7-ADD+)/necrotic (Annexin V-/7-ADD+) NK cells. NC, negative control; MFI, median fluorescence intensity; CT NK, cytotoxic CD56^dim^CD16+ NK. Experiments were measured for eight blood NK-cell samples. Data are presented as mean ± SD. *P < 0.05, **P < 0.01, ***P < 0.001.

To confirm that the IL-6/IL-21/SOCS2/STAT5 is responsible for NK exhaustion in MG, we used the STAT5 activator IL-2 (20 ng/mL, 72h) and siSOCS2 (150 nM, 48h) to interfere with the pathway. Representative flow dot-plots of NK cells after culture with siSOCS2 and/or IL-2 are shown in **Supplementary Figures S5**,**S6**. Co-treatment with siSOCS2 and IL-2 reversed changes of ΔMFI of NKG2D and PD1 by IL-6/IL-21 stimulation (100 ng/mL/50ng/mL, 72h) (**Figures 7A, B**). Similarly, the cytotoxicity (perforin, granzyme B, CD107a production) and ability to secrete IFN-γ of NK cells were recovered in the presence of siSOCS2 and IL-2 (**Figures 7C–E**). The pro-apoptotic effect of IL-6/IL-21 was dramatically higher in total NK cells and was also reversed on the co-intervention of siSOCS2 and IL-2 (**Figures 7F, G**).

## 4 Discussion

In this study, we demonstrated that MGFA II-IV patients are accompanied by strikingly impaired cytokine-secreting ability and cytotoxicity of NK cells. We further investigated the depletion mechanisms of NK cells with a focus on the synergistic inhibition of IL-6/IL-21.

Both the absolute counts and percentages of total NK cells and CT NK cells decreased in MGFA II-IV patients, accompanied by the relative expansions of monocytes and Tfh cells. The changes in the percentages of major immune subsets were more evident than in the absolute numbers for MG patients. The results from correlation analysis revealed that the decrease in the percentages of NK subsets might be due to the expansion of Tfh cells. Tfh cells facilitate the differentiation of plasma cells and affinity maturation of antibodies in MG via an IL-6/IL-21-dependent manner (27, 28). Impaired regulation of Tfh and antibody-producing B cells may be linked to the observed defects in NK function in autoimmune patients, necessitating further investigation (29).

NK-cell activation in MGFA II-IV patients was found to be suppressed, with downregulation of the activating receptor NKG2D and increased expression of the inhibitory receptor PD1. NK-cell cytotoxicity is also impaired in our MGFA II-IV group, manifested as a decline in perforin, granzyme B, CD107a, and K562 cell killing of total NK cells and CT NK cells. A failure of NK cells to kill the autoreactive immune cells may contribute to autoimmune responses (7). NK cells and CD56^bright^ NK cells secrete IFN-γ to play an immunomodulatory role to control auto-reactive inflammation (29, 30). We discovered total NK cells and CS NK cells in MGFA II-IV patients have an impaired ability to produce IFN-γ and could be restored by co-stimulation with IL-6/IL-21.

Many studies have shown that the secretion of IL-6 mainly emanates from the monocytes and favors the accumulation of Tfh cells (31, 32). In patients with systemic juvenile idiopathic arthritis, high levels of IL-6 inhibit the cytotoxicity of NK cells and down-regulate the expression of granzyme B and perforin (15). IL-6 reduced the NK cell-mediated cytotoxicity in castration-resistant prostate cancer by alteration of PD1/NKG2D levels (33). Our results demonstrated that IL-6/SOCS2 upregulated PD1 and downregulated NKG2D and was associated with the exhaustion phenotypes of NK cells. IL-21 contributes to the pathogenesis of autoimmune disorders and has been suggested as a therapeutic target for SLE, TID, rheumatoid arthritis, etc (34). IL-21 is critical to Tfh cell development and participates in a positive feedback loop on CD4+ T cells (35, 36). Previous studies identified that IL-21 favors the proliferation and activity of human NK cells (37). In the absence of IL-2, IL-21 showed no significant effect on the expression of a panel of surface receptors (38). Furthermore, the high concentration of IL-21 significantly promoted apoptosis in NK cells via activating caspases 8-10 (24). The above findings were in accordance with our results.

SOCS2 protein is an important negative regulator of various cytokine signalings (39). Studies have shown that both IL-6 and IL-21 can upregulate and activate the expression of SOCS family proteins (40, 41). SOCS2 restricted NK-cell differentiation and development via inhibiting direct interaction with JAK2/STAT5 (42). In the present study, we investigated the impact of IL-6/IL-21 synergistic stimulation on SOCS2/STAT5 regulation in NK cells from individuals with MG. While IL-2, a known activator of STAT5 is necessary for NK cells to initiate priming, proliferation, and boost effector function. IL-2 is relatively deficient in severe MG (43), although not statistically significant in our study. Defects in IL-2 signaling fail to maintain self-tolerance and induce IFN-γ production by NK cells (44). Our results indicated that exhausted NK cells in MG could be reinvigorated by IL-2 supplementation and SOCS2 inhibition.

The subsequent enrichment analysis showed that DEGs were also related to the IgA immunoglobulin complex and IL-17 signaling pathway. IL-17A constrained NK-cell antitumor and antiviral activity via the inhibitory function of SOCS3 on IL-15 signaling (14). Both secretory IgA and dimeric IgA proteins mediated the inhibition of NK activity by interaction with FcαR on the NK-cell surface (45). Further studies on the above-described molecular mechanisms involved in the regulation of NK cells will provide novel approaches for NK-cell therapy in MG. The functionality and cellular longevity of NK cells have been reported to be restricted by IL-8 and IL-17 (14, 23). It will be interesting for future work to investigate the impact of the MG-related cytokine storm on NK cells in different stages of MG development.

This exploratory study has several limitations that warrant consideration. Firstly, no primary immune cell line was used in the NK co-culture assay apart from the K562 cells, this proposition will need to be verified. Secondly, Further work will be required to elucidate whether the NK-cell exhaustion is an etiologic contributor to MG exacerbation or just a consequence of the expansion of monocytes and Tfh cells. Thirdly, single-cell sequencing and spatial transcriptome analysis will provide direct evidence to determine which phenotypes of NK cells promote versus inhibit MG and are worth to be applied in the future.

**Figure 8:**
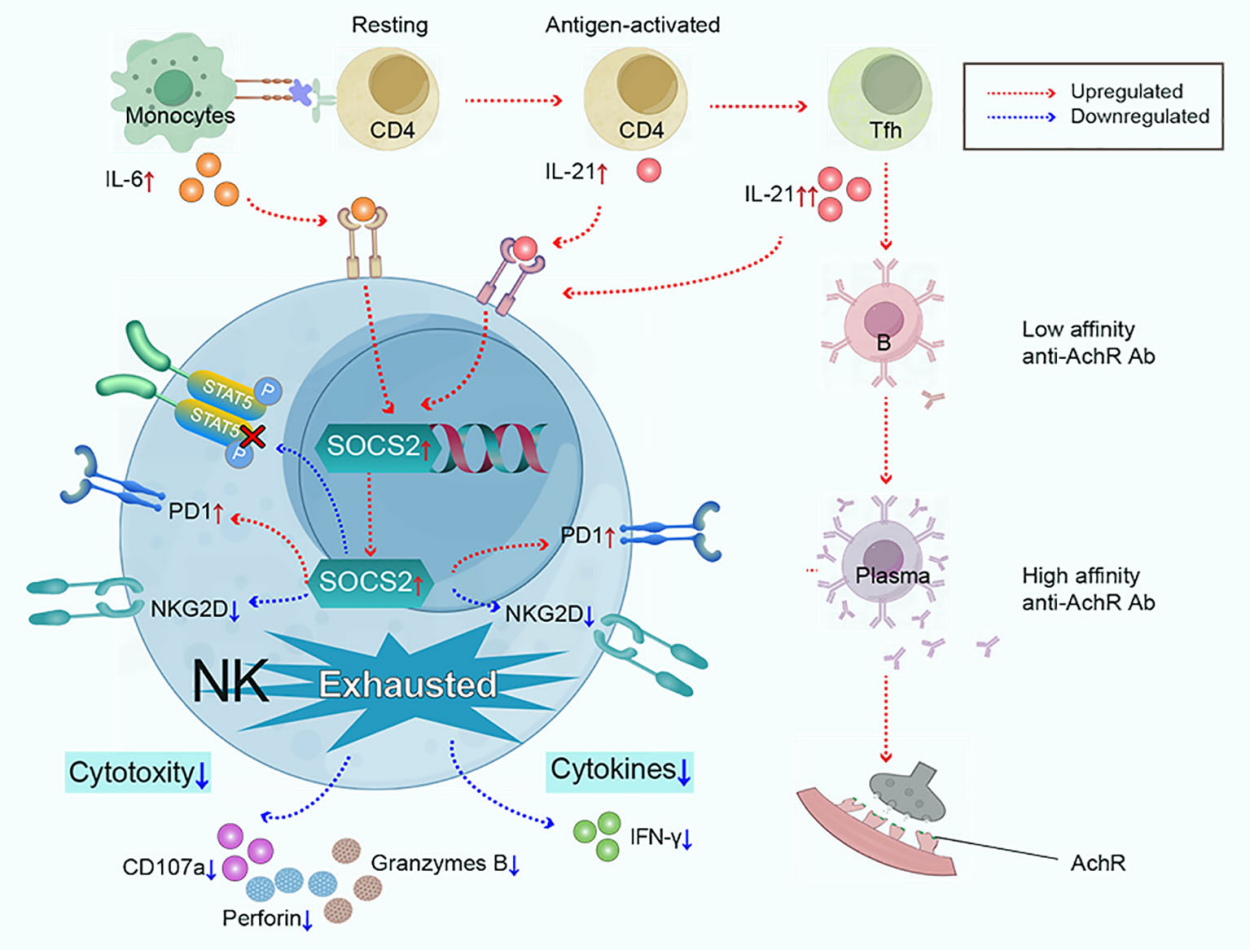
Monocytes and Tfh cells greatly expand in myasthenia gravis, leading to the large secretion of IL-6 and IL-21. IL-6 and IL-21 work cooperatively to mediate NK-cell exhaustion by up-regulating SOCS2 and impairing the phosphorylation of STAT5. Subsequently, NK cells exhibit exhausted signature, characterized by low cytokine production (IFN-γ), decreased cytotoxicity (perforin, granzyme B, and CD107a), downregulated activating receptor NKG2D, and upregulated inhibitory receptor PD1.

In summary, our findings highlight exhausted NK phenotypes are key features in the immunopathology of MG, characterized by impaired cytotoxicity and IFN-γ section, low expression of NKG2D, and high expression of PD1. We propose that the synergy effect of IL-6/IL-21 mediates NK-cell exhaustion by upregulating SOCS2 and inhibiting the phosphorylation of STAT5 in MG and our study results support this hypothesis (**Figure 8**). Therefore, targeting the IL-6/IL-21/SOCS2/STAT5 pathways to restore NK-cell function against autoimmunity may hold therapeutic promise for MG.

## Data Availability

The names of the repository/repositories and accession number(s) can be found below: https://www.ncbi.nlm.nih.gov/bioproject/PRJNA1035806.
