# Supplementary material for: Exhausted signature and regulatory network of NK cells in myasthenia gravis
Source: Front Immunol. 2024 Sep 13;15:1397916. doi: 10.3389/fimmu.2024.1397916 (PMC11427316; doi:10.3389/fimmu.2024.1397916)
Supplement: Supplementary file 1 [file DataSheet1.docx]

Supplementary Material

Exhausted signature and regulatory network of NK cells in myasthenia gravis

Qing Zhang^1,2^, Xingyu Han^1,2^, Zhuajin Bi^1,2^, Mengge Yang^1,2^, Jing Lin^1,2^, Zhijun Li^1,2^*, Min Zhang^1,2^*, Bitao Bu^1,2^*

^1^ Department of Neurology, Tongji Hospital, Tongji Medical College, Huazhong University of Science and Technology, Wuhan, 430030, China

^2^ Hubei Key Laboratory of Neural Injury and Functional Reconstruction, Huazhong University of Science and Technology, 430030 Wuhan, China

*** Correspondence:**Corresponding Author
lizhijun@tjh.tjmu.edu.cn (Zhijun Li); zhang_min_3464@126.com (Min Zhang); bubitao@tjh.tjmu.edu.cn (Bitao Bu)

# Supplementary Table S1.

**TABLE S1. Primers used in the RT-qPCR to amplify the coding region.**

| Genes | Forward sequences (from 5′ to 3′) | Reverse sequences (from 5′ to 3′) | Fragment size |
| --- | --- | --- | --- |
| S100A8 | GTTCTGTTTTTCAGGTGGGGC | CGTCTGCACCCTTTTTCCTGA | 193 |
| S100A9 | TCCTCGGCTTTGACAGAGTG | TGCCCCAGCTTCACAGAGTA | 110 |
| FHL3 | AGTCCCTGTATGGACGCAAG | TCTTCATAGAACAGCTCCCTCG | 139 |
| JCHAIN | TGTACCATTTGTCTGACCTCTGT | GCAGGTCTCTGTAGCACTGT | 119 |
| SOCS2 | GTAGGGTAGAGGTGCCGAGA | TCACCCACTGATCGCCTGC | 142 |
| FCMR | TACTTCCTGCCAGTATCGGG | ACCACGGTACCACATGTTCC | 161 |
| GAPDH | TCGGAGTCAACGGATTTGGT | TGATGACAAGCTTCCCGTTG | 138 |

# Supplementary Figure S1.


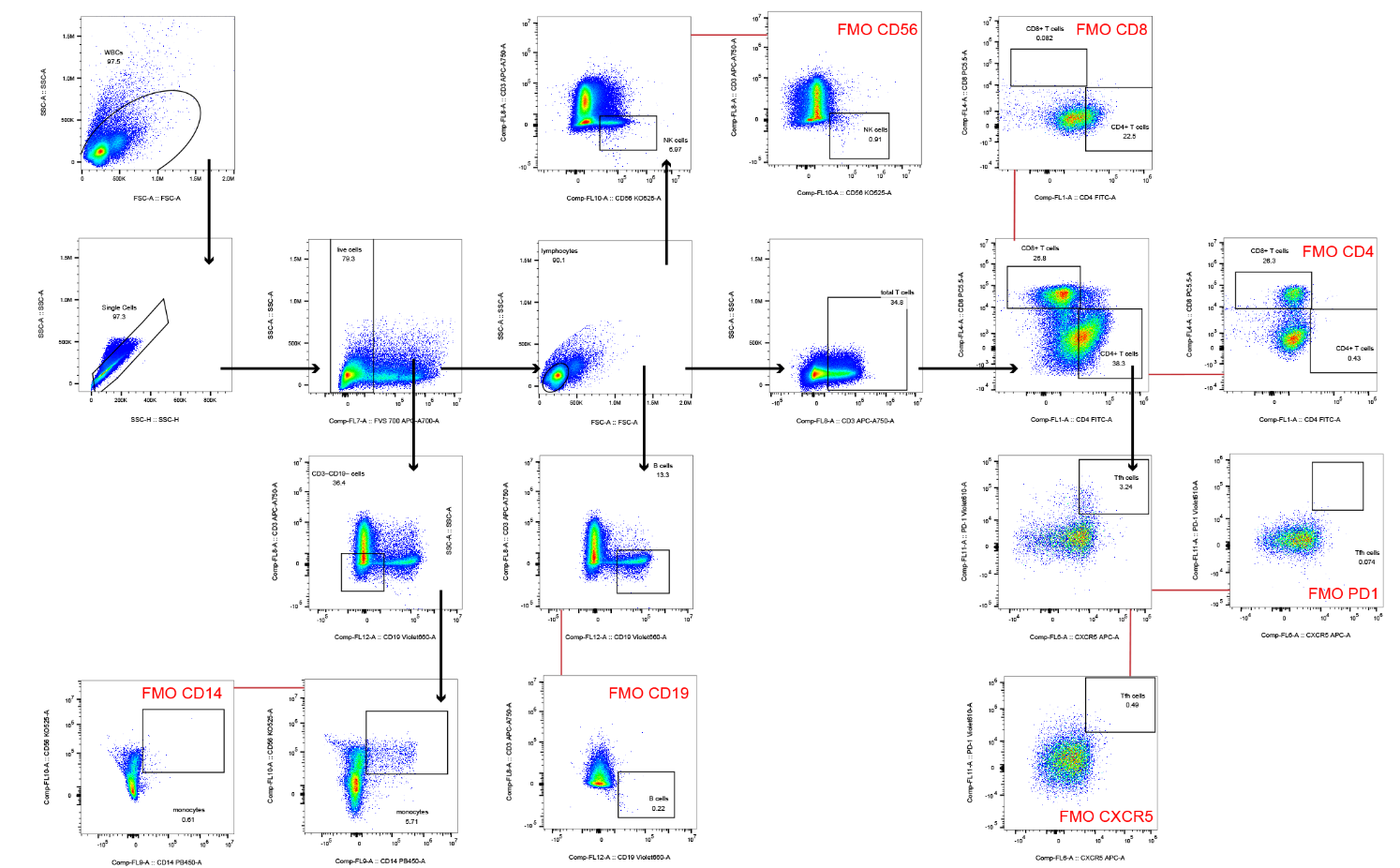


**SUPPLEMENTARY FIGURE S1.** The gating strategy for major immune subsets in the human peripheral blood by flow cytometry.

# Supplementary Figure S2.


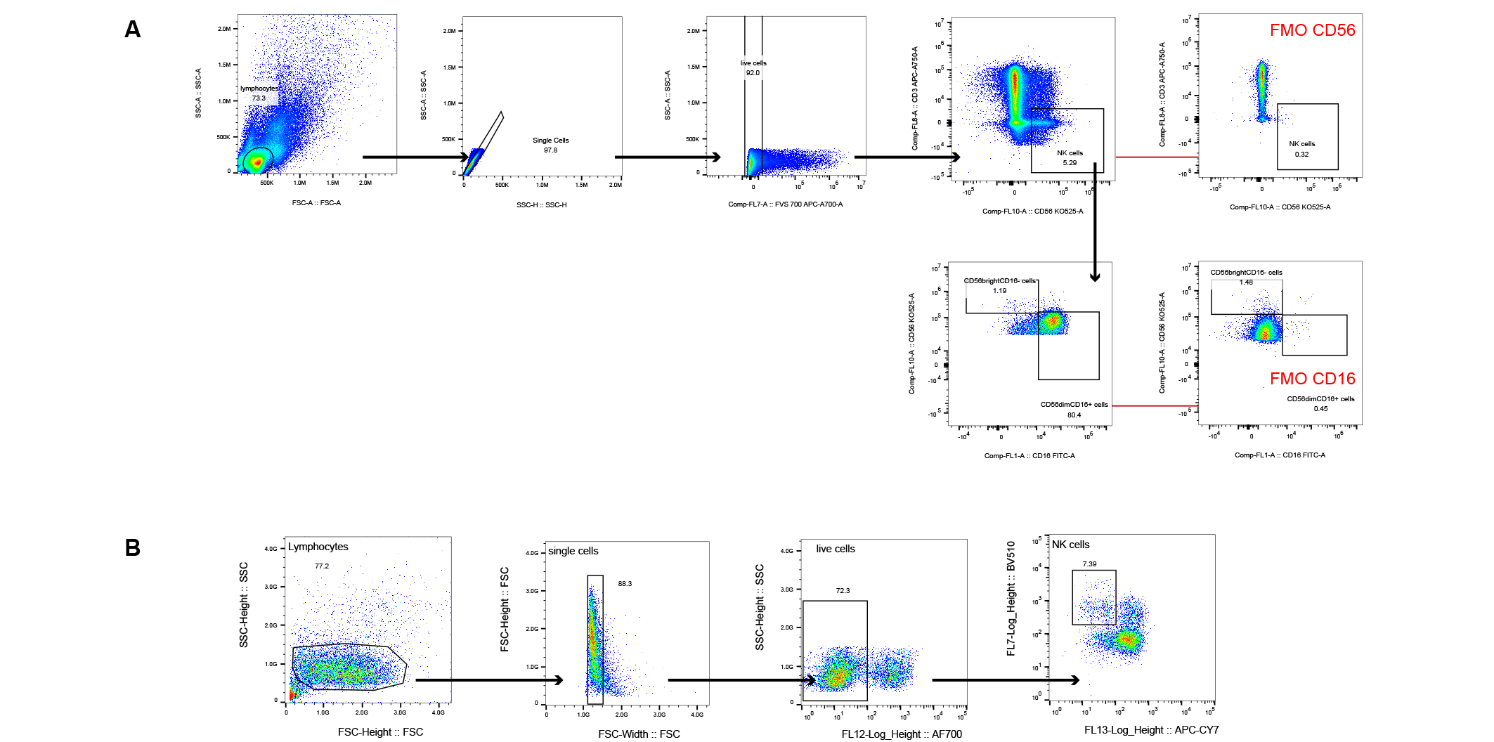


**SUPPLEMENTARY FIGURE S2.** Gating strategy of NK cells. **(A)** Sequential strategy for gating cytotoxic CD56^dim^CD16+ NK (CT NK) cells and cytokine-secreting CD56^bright^CD16− NK (CS NK) cells in the human peripheral blood. **(B)** Diagram of sorting strategy for total NK cells.

# Supplementary Figure S3.


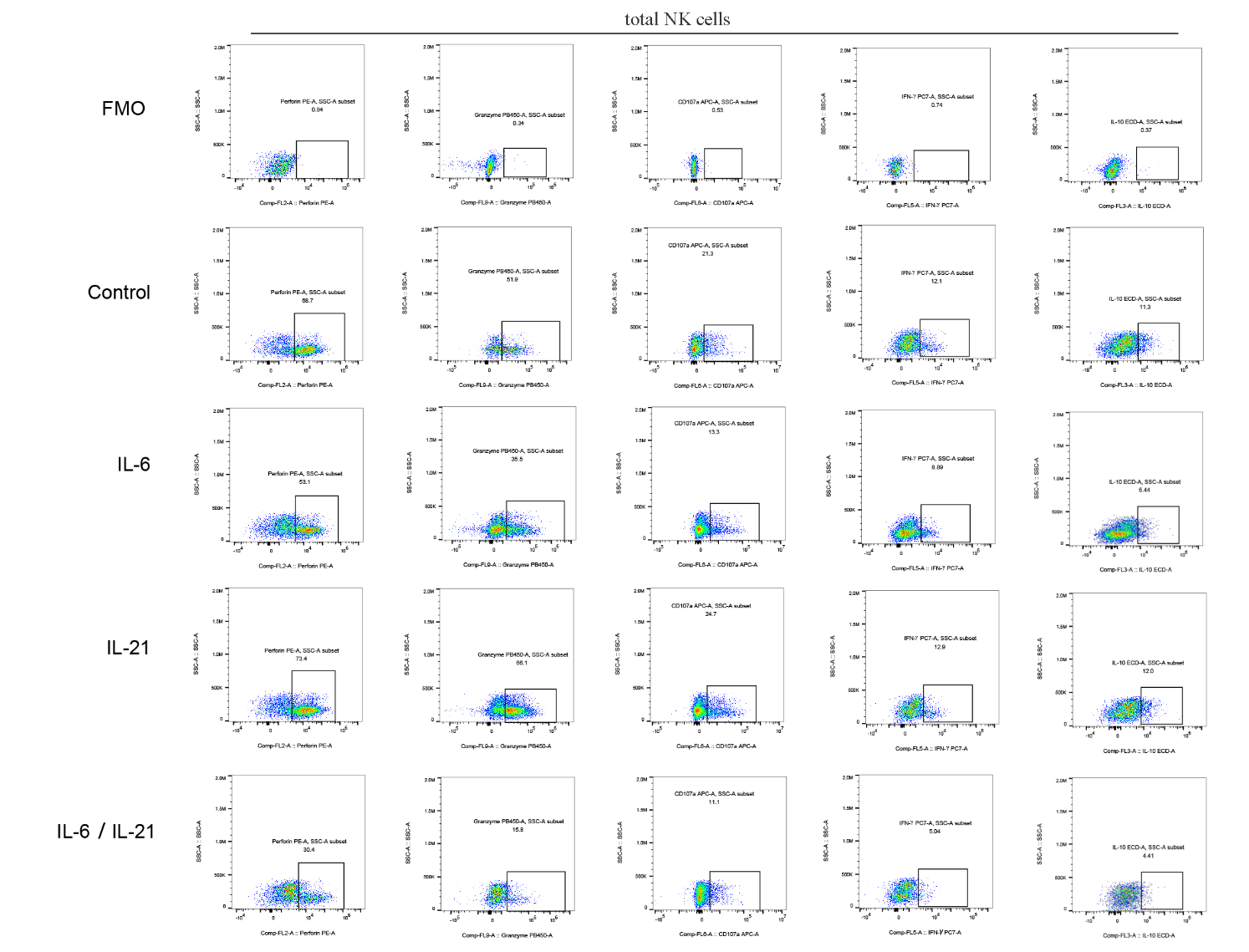


**SUPPLEMENTARY FIGURE S3.** Representative flow cytometry plots showing the frequency of perforin, granzyme B, CD107a, IFN-γ, and IL-10 production by total NK cells between control, IL-6, IL-21, and IL-6/IL-21 group.

# Supplementary Figure S4.


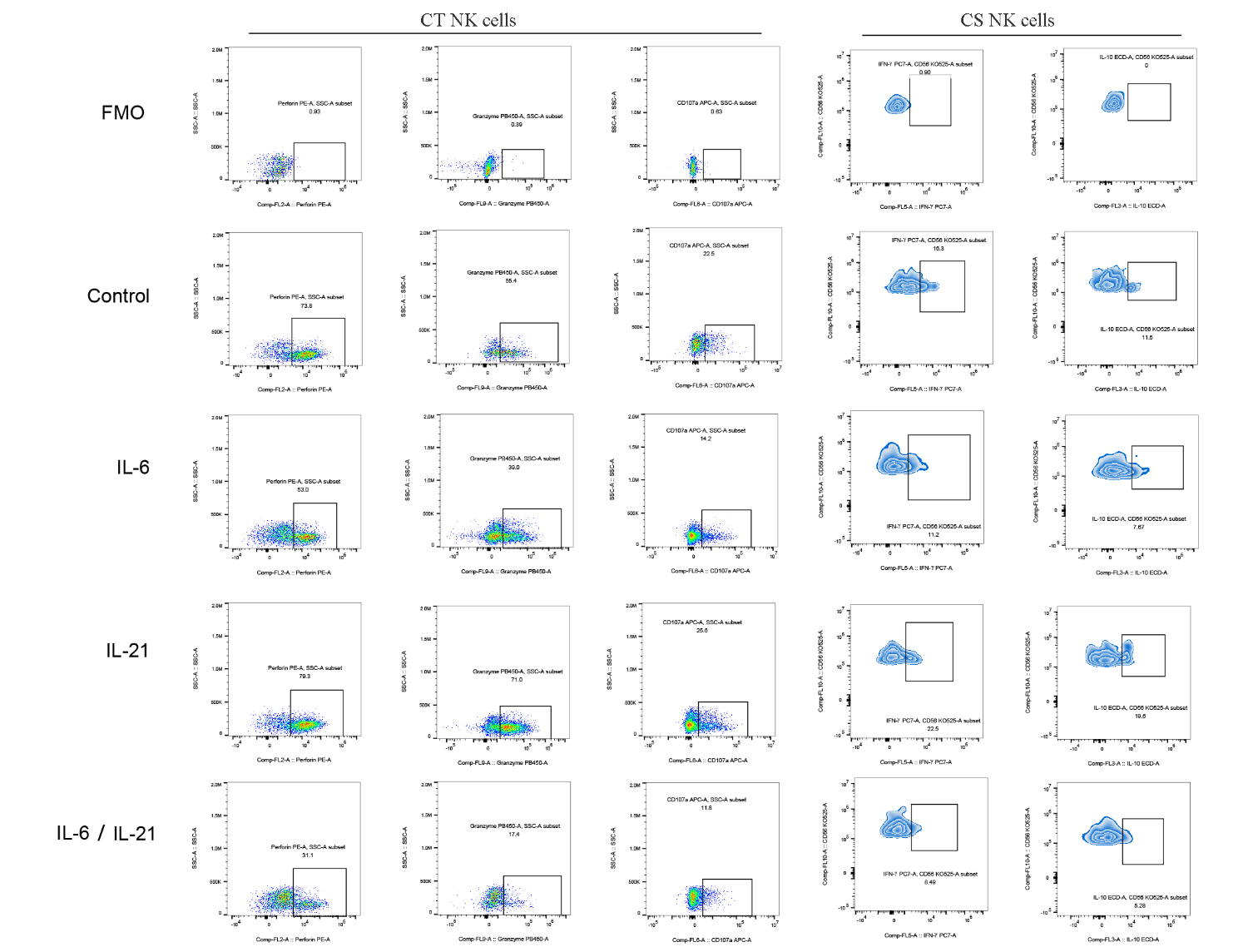


**SUPPLEMENTARY FIGURE S4.** Representative flow cytometry plots showing the frequency of perforin, granzyme B, CD107a by cytotoxic CD56^dim^CD16+ NK (CT NK) cells, and IFN-γ, IL-10 production by cytokine-secreting CD56^bright^CD16− NK (CS NK) cells between control, IL-6, IL-21, and IL-6/L-21 group.

# Supplementary Figure S5.


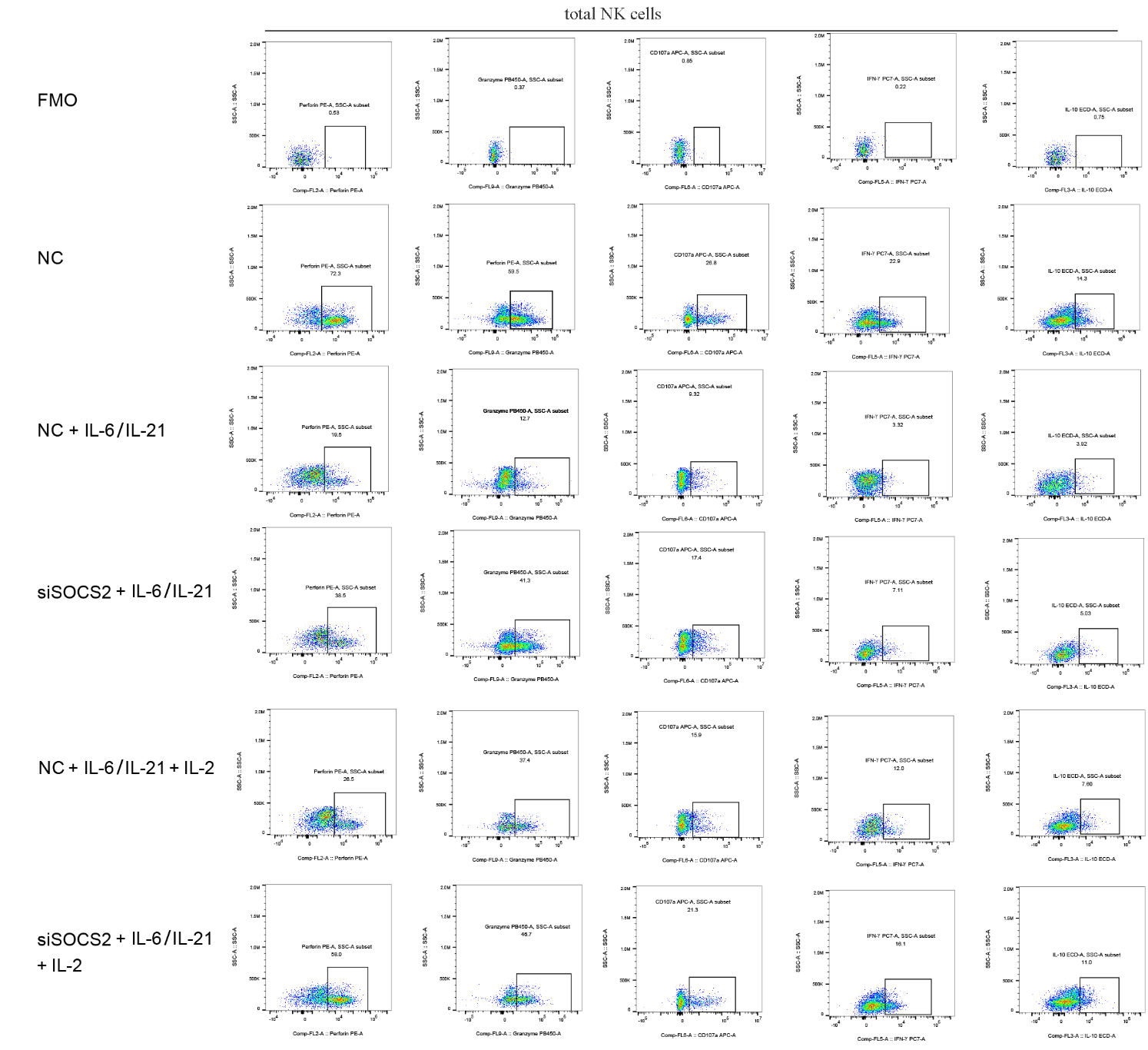


**SUPPLEMENTARY FIGURE S5.** Representative flow cytometry plots showing the frequency of perforin, granzyme B, CD107a, IFN-γ, IL-10 production by total NK cells between NC, NC + IL-6/IL-21, siSOCS2 + IL-6/IL-21, NC + IL-6/IL-21 + IL-2, and siSOCS2+ IL-6/IL-21 + IL-2 group.

# Supplementary Figure S6.


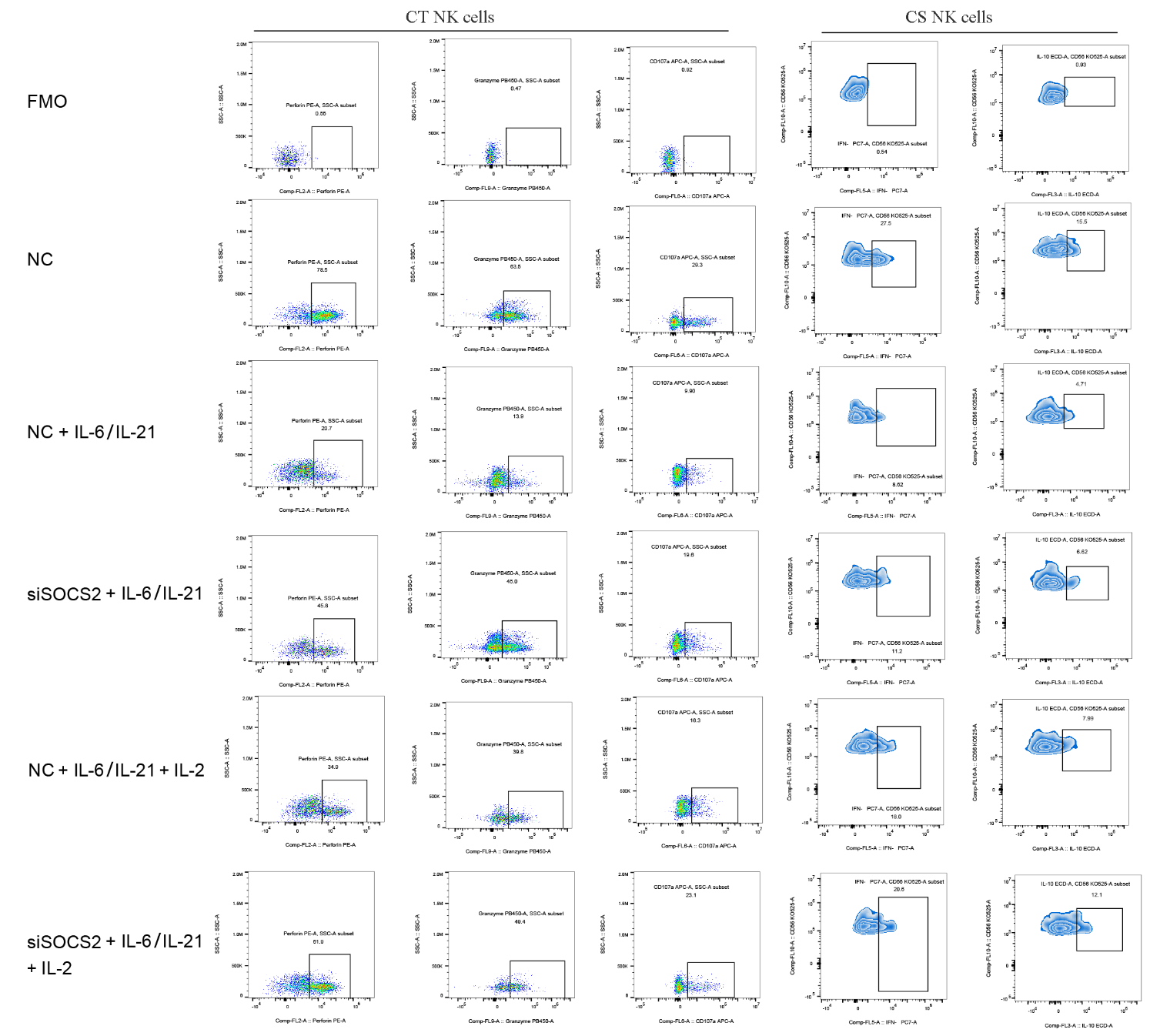


**SUPPLEMENTARY FIGURE S6.** Representative flow cytometry plots showing the frequency of perforin, granzyme B, CD107a by cytotoxic CD56^dim^CD16+ NK (CT NK) cells, and IFN-γ, IL-10 production by cytokine-secreting CD56^bright^CD16− NK (CS NK) between NC, NC + IL-6/IL-21, siSOCS2 + IL-6/IL-21, NC + IL-6/IL-21 + IL-2, and siSOCS2+ IL-6/IL-21 + IL-2 group.
